# Quorum sensing systems differentially regulate the production of phenazine-1-carboxylic acid in the rhizobacterium *Pseudomonas aeruginosa* PA1201

**DOI:** 10.1038/srep30352

**Published:** 2016-07-26

**Authors:** Shuang Sun, Lian Zhou, Kaiming Jin, Haixia Jiang, Ya-Wen He

**Affiliations:** 1 State Key Laboratory of Microbial Metabolism, School of Life Sciences & Biotechnology, Shanghai Jiao Tong University, Shanghai 200240, China

## Abstract

*Pseudomonas aeruginosa* strain PA1201 is a newly identified rhizobacterium that produces high levels of the secondary metabolite phenazine-1-carboxylic acid (PCA), the newly registered biopesticide Shenqinmycin. PCA production in liquid batch cultures utilizing a specialized PCA-promoting medium (PPM) typically occurs after the period of most rapid growth, and production is regulated in a quorum sensing (QS)-dependent manner. PA1201 contains two PCA biosynthetic gene clusters *phz1* and *phz2*; both clusters contribute to PCA production, with *phz2* making a greater contribution. PA1201 also contains a complete set of genes for four QS systems (LasI/LasR, RhlI/RhlR, PQS/MvfR, and IQS). By using several methods including gene deletion, the construction of promoter-*lacZ* fusion reporter strains, and RNA-Seq analysis, this study investigated the effects of the four QS systems on bacterial growth, QS signal production, the expression of *phz1* and *phz2*, and PCA production. The possible mechanisms for the strain- and condition-dependent expression of *phz1* and *phz2* were discussed, and a schematic model was proposed. These findings provide a basis for further genetic engineering of the QS systems to improve PCA production.

The phenazines are nitrogen-containing, colored, aromatic, secondary metabolites produced by various fluorescent pseudomonads, streptomycetes, and members of a few other bacterial genera^1^,^2^. These compounds are best known for their antibiotic properties, and affect a broad spectrum of organisms including bacteria, fungi, plants, nematodes, parasites, and humans^3^,^4^. Phenazines are also involved in numerous aspects of bacterial physiology such as survival, iron acquisition, signaling, and biofilm formation, and they have been studied extensively as microbial fitness determinants^5^,^6^,^7^,^8^. The pseudomonads, particularly *Pseudomonas aeruginosa*, *P*. *chlororaphis*, and *P*. *fluorescens*, are well-studied examples of phenazine producers. They share a common operon (*phz*ABCDEFG) which encodes the enzymes needed for the synthesis of PCA, a precursor to the other phenazines^9^. All *P*. *aeruginosa* strains contain the two nearly identical PCA biosynthetic gene clusters *phzA*_*1*_*B*_*1*_*C*_*1*_*D*_*1*_*E*_*1*_*F*_*1*_*G*_*1*_ (abbreviated as *phz1*) and *phzA*_*2*_*B*_*2*_*C*_*2*_*D*_*2*_*E*_*2*_*F*_*2*_*G*_*2*_ (abbreviated as *phz2*). Both clusters contribute to phenazine production^7^,^10^,^11^. In addition, some phenazine-producing bacteria also contain one or more accessory genes such as *phzO*, *phzH*, *phzM*, and *phzS*, that encode different terminal-modifying enzymes for conversion of PCA into additional phenazine derivatives such as 2-hydroxyphenazine (2OH-PCA), phenazine-1-carboxamide (PCN), and pyocyanin (PYO)^12^,^13^,^14^,^15^. Phenazine production is controlled by complex regulatory networks. Environmental parameters such as temperature, pH, salinity, oxygen or nutrient availability have been shown to affect phenazine biosynthesis^7^,^16^,^17^,^18^. Membrane sensor proteins and two-component sensors control the activity of downstream regulators such as quorum sensing systems, RNA-binding proteins and small RNAs; these cytoplasmic regulators then directly or indirectly control the expression of the *phz* clusters^17^,^19^,^20^,^21^,^22^,^23^.

*P. aeruginosa* strains contain four QS systems: two acyl-homoserine lactone (HSL)-based QS systems, the quinolone-based QS system, and the newly identified IQS-dependent QS system^24^,^25^. In the acyl-HSL-based QS systems, two acyl-HSL synthase enzymes LasI and RhlI are responsible for the synthesis of N-3-oxo-dodecanoyl homoserine lactone (abbreviated as 3-oxo-C12-HSL) and N-butanoyl-homoserine lactone (abbreviated as C4-HSL), respectively. 3-oxo-C12-HSL and C4-HSL can form a complex with the transcriptional regulators LasR and RhlR to regulate the expression of downstream target genes^26^,^27^,^28^. In the quinolone-dependent QS system, PqsABCDE and PqsH are involved in the synthesis of 2-heptyl-4-quinolone (abbreviated as HHQ) and 2-heptyl-3-hydroxy-4-quinolone (abbreviated as PQS)^29^. Both HHQ and PQS can bind to the transcriptional regulator MvfR and form a complex to regulate gene expression^30^. It was originally proposed that the newly identified QS signal IQS [2-(2-hydroxyphenyl)-thiazole-4- carbaldehyde]^24^ is synthesized via the gene cluster *ambBCDE*^24^. However, recent results with *P. aeruginosa* strongly suggest that IQS is a byproduct from the biosynthesis of the siderophore pyochelin^31^. The cluster *ambBCDE* is responsible for the production of L-2-amino-4-methoxy-*trans*-3-butenoic acid (AMB), a non-proteinogenic amino acid^32^. The IQS receptor remains elusive. The mechanism whereby these QS systems control expression of *phz1* and *phz2*, and the mechanisms that confer differential, condition-dependent expression of *phz1* and *phz2*, have not been thoroughly characterized. For example, although their genomes share high sequence similarity, the *P*. *aeruginosa* strains PAO1, PA14, and M18 exhibit strain-dependent differences with respect to QS-dependent regulation of phenazine production. In the clinical isolates PAO1 and PA14, Las- and Rhl-defective mutant strains lose the ability to produce PYO^33^, while in the rhizosphere strain M18, the Las and Rhl systems are apparently negative regulators of phenazine production^34^. The mechanisms underlying these activities still need further elucidation at a molecular level. Also, the pattern of QS-dependent *phz* gene expression may depend on culture conditions. Under aerobically grown, well-mixed planktonic culture conditions, PQS is required for *phz1* expression and *phz1* is a major contributor to phenazine biosynthesis in *P*. *aeruginosa* PA14. However, when PA14 is grown as a colony biofilm on agar plates, *phz2* alone is sufficient for wild-type phenazine production^7^. Finally, how the newly identified IQS signal affects PYO biosynthesis is still not known.

Due to rising concerns in the 1980s about the use of chemical pesticides, researchers became very interested in the antifungal properties of phenazines produced by soil pseudomonads and their potential use in the control of phytopathogenic fungi. *Pseudomonas aeruginosa* M18 is an effective biocontrol agent which was isolated from the rhizosphere of sweet melon^35^. The predominant phenazine produced by M18 is phenazine-1-carboxylic acid (PCA) (Fig. 1a). After several rounds of genetic modifications, the PCA yield of the genetically modified strain M18UMS/Phz reached approximately 4.7 g/L^36^,^37^. PCA was commercially named shenqinmycin, and a 1% shenqinmycin suspension was registered in China as an environmentally friendly fungicide (Product no. PD20110315) in 2011. This product is being marketed in China to control rice and vegetable diseases caused by *Rhizoctonia solani* and *Fusarium oxysporum*^38^.

*P. aeruginosa* strain PA1201 was originally isolated from the rice rhizosphere, and displayed strong inhibitory activity towards the pathogens *R. solani* and *Xanthomonas oryzae* pv. *oryzae*^39^. The PCA yield of strain PA1201 was originally higher than that of M18 and PA1201 was shown to have more biotechnological potential for industrial production of PCA^39^,^40^. In this study, we clone and analyze the genes for the four known QS systems, for PCA biosynthesis, and for PCA modification in PA1201. The relative contribution of the two *phz* clusters to PCA production is analyzed. The effects of the four QS systems on bacterial growth, QS signal production, the expression of the two *phz* clusters, and PCA biosynthesis is investigated in a specialized PCA promoting medium (PPM). These findings provide a basis for further genetic engineering of the QS systems to improve PCA production in PA1201.

## Results

### Genomic analysis of the genes for the four QS systems and PCA biosynthesis in PA1201

**Figure 1:**
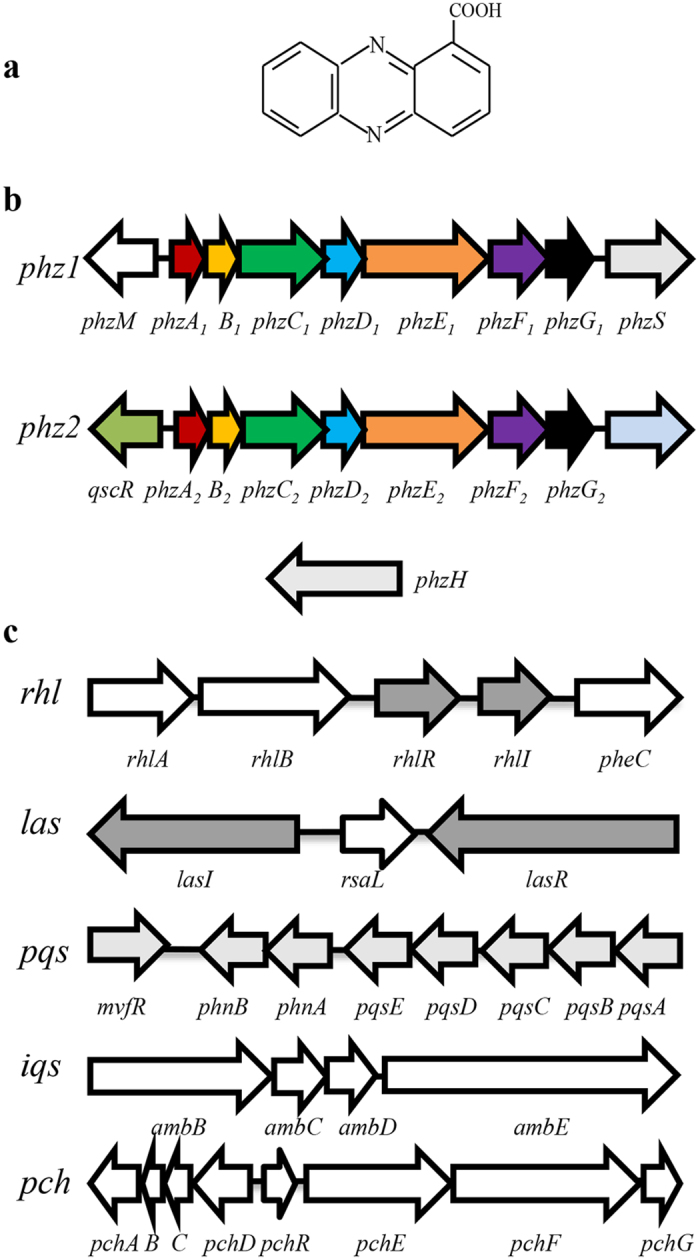
PCA biosynthetic and quorum sensing gene clusters in PA1201. (**a**) The chemical structure of phenazine-1-carboxylic acid, (**b**) Organization of the PCA biosynthetic gene clusters, and (**c**) Gene clusters for four quorum-sensing systems in *P. aeruginosa* strain PA1201.

Based on the genome sequence of *P. aeruginosa* strain PA14, several primer pairs were designed to clone the genes for the four QS systems and PCA biosynthesis using PCR amplification in strain PA1201. Subsequent sequence analysis confirmed that strain PA1201 has two PCA biosynthetic gene clusters, *phz1* and *phz2* (Fig. 1b). Strain PA1201 also contained three functional accessory genes, *phzM*, *phzS*, and *phzH*, which encode the enzymes that convert PCA into PYO and PCN, and the genes for the three classic QS systems, i.e., the 3-oxo-C12-HSL-dependent LasI/LasR system, the C4-HSL-dependent RhlI/RhlR system, and the PQS-dependent PqsABCDE/MvfR system (Fig. 1c). In addition, the *amb* gene cluster for AMB biosynthesis and the *pch* gene cluster for pyochelin biosynthesis were also identified in PA1201 (Fig. 1c). The genomic organization of these genes in strain PA1201 was essentially identical to that of *P. aeruginosa* strains PAO1, PA14, and M18. The products of these strain PA1201 genes showed >99% amino acid identity with their counterparts in the other *P. aeruginosa* strains. DNA sequences of all above genes are available in NCBI Database (KX173291-173307).

### The effects of four QS systems on PCA production in strain MSH

**Figure 2:**
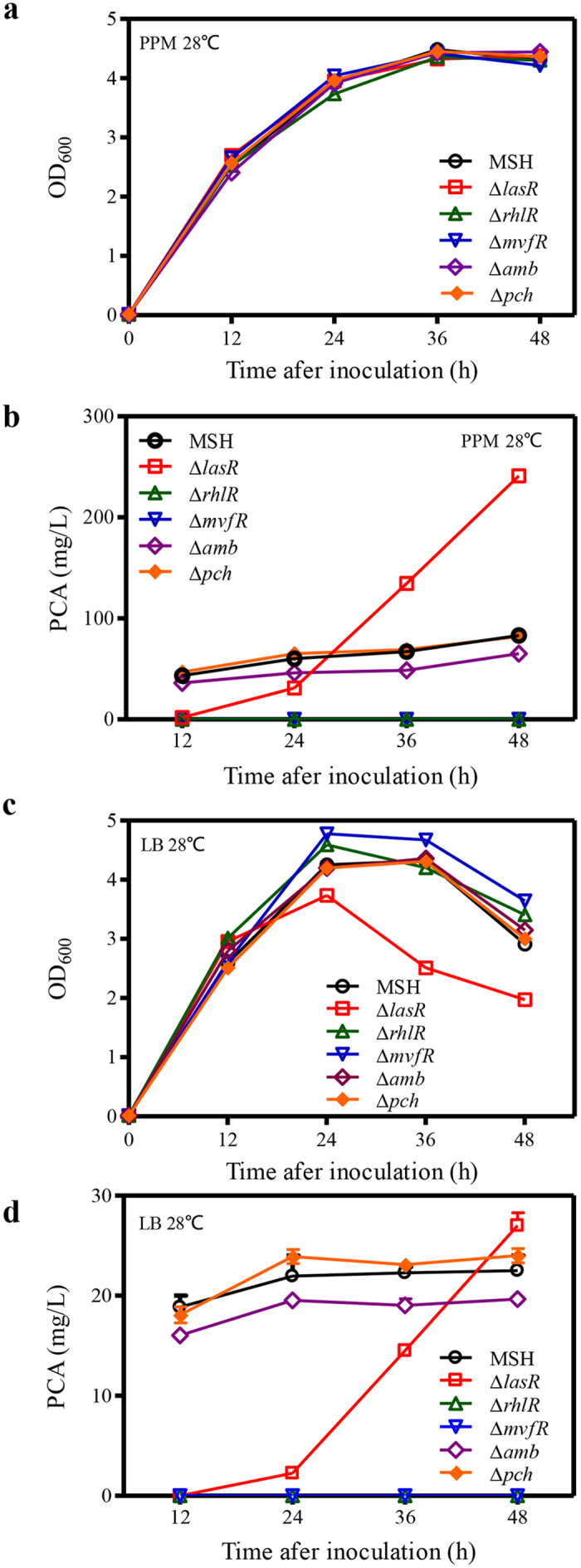
PCA production of PA1201-derived strains in fed-batch cultures. (**a**) Growth of PA1201-derived strains in PPM medium. (**b**) PCA production during growth in PPM medium. (**c**) Growth of PA1201-derived strains in LB medium. (**d**) PCA production during growth in LB medium. Values shown are the means ± one SD from three independent experiments.

The presence of *phzM*, *phzS*, and *phzH*, enables strain PA1201 to produce two additional phenazines, PCN and PYO^39^. To accurately assess the potential of PCA production in strain PA1201, *phzM*, *phzS*, and *phzH* were all deleted and the resulting strain MSH produced only PCA during fermentation (Supplementary Fig. S1). The genes *lasR*, *rhlR*, *mvfR*, and gene clusters *amb* and *pch* were each individually, *in-frame* deleted from strain MSH using the strategy shown in Supplementary Fig. S2a. The resulting single deletion mutant strains Δ*lasR*, Δ*rhlR*, Δ*mvfR*, Δ*amb*, and Δ*pch* were analyzed for growth and PCA production. Individual deletion of each of these genes or gene clusters had little effect on bacterial growth in PPM medium (Fig. 2a). We observed that deletion of *rhlR* or *mvfR* abolished PCA production during growth in PPM medium, but deletion of either the *amb* or *pch* gene clusters had little effect on PCA production (Fig. 2b). In contrast, the regulator LasR displayed differential regulation of PCA production. PCA levels of Δ*lasR* were significantly lower than those of MSH during the logarithmic growth phase (12–24 h), but significantly higher during stationary phase (36–48 h) (Fig. 2b).

The growth and PCA production of the above strains were also determined in LB medium. Although strains Δ*rhlR*, Δ*mvfR*, and Δ*amb* again displayed growth patterns similar to those of strain MSH (Fig. 2c), strain Δ*lasR* grew more slowly during late log phase and declined more rapidly during stationary phase than strain MSH (Fig. 2c). The PCA production patterns of strains Δ*lasR*, Δ*rhlR*, Δ*mvfR*, Δ*amb*, and Δ*pch* in LB medium were similar to what was observed in PPM medium (Fig. 2d); therefore, PPM medium was used to evaluate PCA production in subsequent experiments.

### The relative contributions of the two *phz* clusters to PCA production

**Figure 3:**
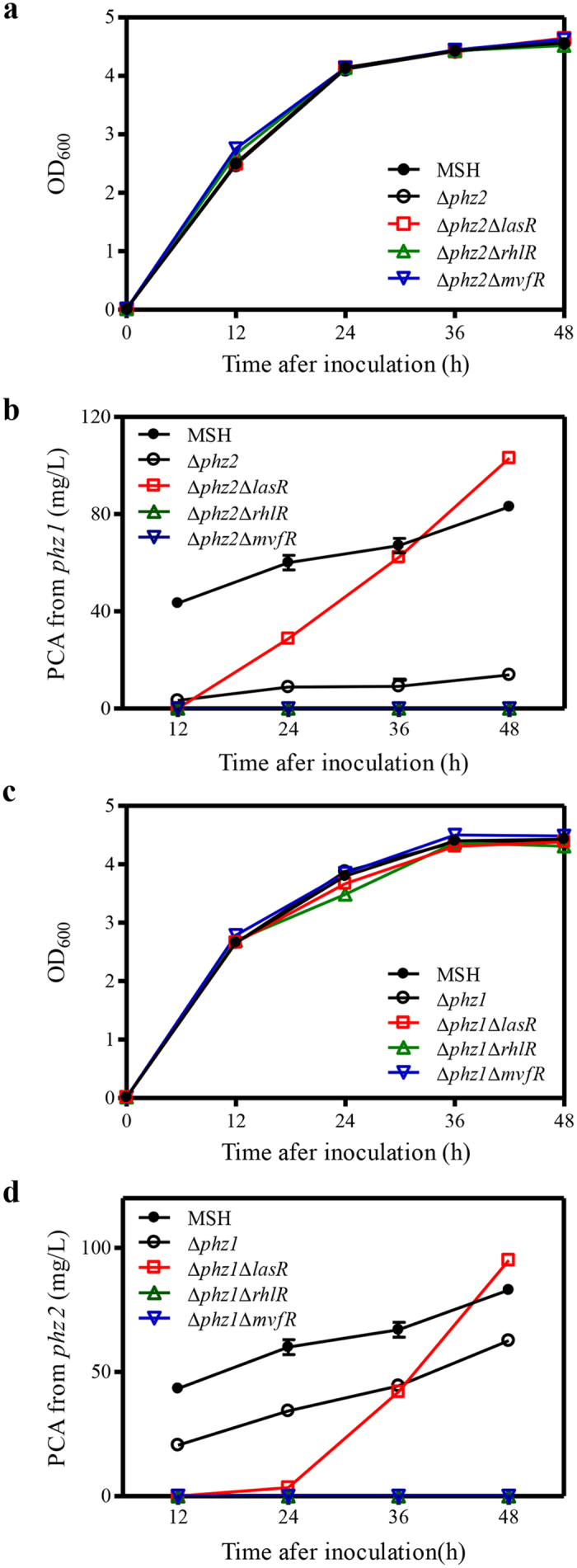
Time course of PCA production in PA1201-derived strains. (**a**) Growth of PA1201-derived strains in PPM medium. (**b**) PCA production of strains MSH, Δ*phz2*, Δ*phz2*Δ*lasR*, Δ*phz2*Δ*rhlR*, and Δ*phz2*Δ*mvfR*. (**c**) Growth of PA1201-derived strains in PPM medium. (**d**) PCA production of strains MSH, Δ*phz1*, Δ*phz1*Δ*lasR*, Δ*phz1*Δ*rhlR*, and Δ*phz1*Δ*mvfR*. Values shown are the means ± one SD from three independent experiments.

PA1201 contains two PCA biosynthetic gene clusters: *phz1* and *phz2* (Fig. 1b). To investigate the relative contributions of *phz1* and *phz2* to PCA biosynthesis in PA1201, we deleted each *phz* cluster individually in the strain MSH. The resulting strains, Δ*phz1* and Δ*phz2* showed growth patterns which were nearly identical to that of the parent strain MSH (Fig. 3a,c). Quantification of PCA levels in culture supernatants revealed that both strains Δ*phz1* and Δ*phz2* produced significantly less PCA than the parent strain MSH. The PCA levels from *phz1* (Δ*phz2*) were only ten to twenty percent of those observed with MSH (Fig. 3b). In contrast, the PCA levels from *phz*2 (Δ*phz1*) were approximately seventy percent of those observed with MSH (Fig. 3d). These results suggest that both *phz* clusters contribute to PCA production, with *phz2* making a greater contribution.

### Effects of three QS systems on *phz1*- or *phz2*-dependent PCA production

Since both *phz1* and *phz2* contributed significantly to PCA production in strain PA1201 (Fig. 3), we reasoned that the three QS systems might have effects on both gene clusters. To determine the effects of the three QS systems on *phz1*-dependent PCA production, we generated the double mutation strains Δ*phz2*Δ*lasR*, Δ*phz2*Δ*rhlR*, and Δ*phz2*Δ*mvfR*. These strains were nearly identical in growth to strain Δ*phz2* (Fig. 3a). Deletion of *rhlR* or *mvfR* abolished *phz1*-dependent PCA production (Fig. 3b). Deletion of *lasR* had no significant effect on *phz1*-dependent PCA production at 12 h; however, it significantly increased *phz1*-dependent PCA production at 24–48 h after inoculation (Fig. 3b).

To study the effects of the three QS systems on *phz2*-dependent PCA production, the genes *lasR*, *rhlR*, and *mvfR* were individually deleted from strain Δ*phz1*, and these double mutation strains were nearly identical in growth to strain Δ*phz1* (Fig. 3c). When we compared these strains Δ*phz1*Δ*lasR*, Δ*phz1*Δ*rhlR*, and Δ*phz1*Δ*mvfR* to strain Δ*phz1* for PCA production, we again found that deletion of *rhlR* and *mvfR* abolished *phz2*-dependent PCA production. Deletion of *lasR* resulted in a reduction in *phz2*-dependent PCA production during 12–24 h and a significant increase at 36 to 48 h after inoculation (Fig. 3d).

### Production of 3-oxo-C12-HSL, C4-HSL and PQS in the QS mutants

**Figure 4:**
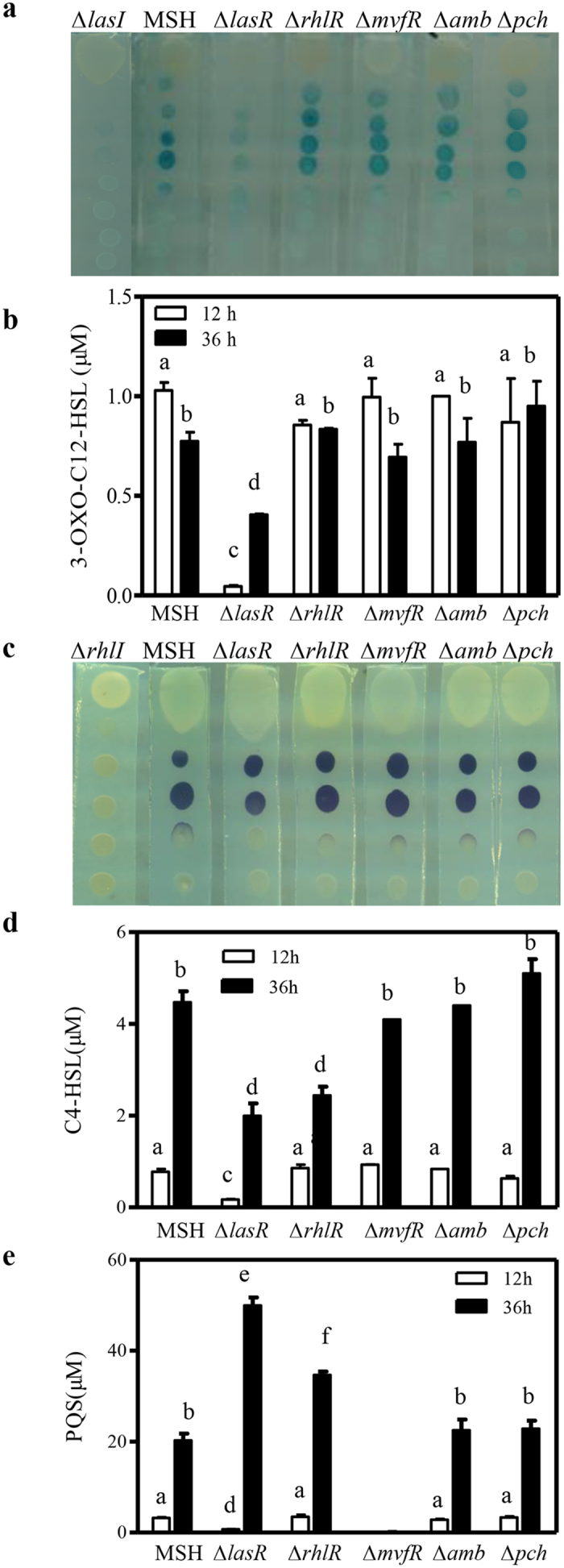
QS signal production of PA1201-derived strains. (**a**) Bioassay of 3-oxo-C12-HSL production using the reporter strain CF11. Blue spots indicate that the diffusible signal was detected by the reporter cells. (**b**) LC-MS analysis of 3-oxo-C12-HSL levels of PA1201-derived strains at 12 and 36 h after inoculation in PPM medium. (**c**) Bioassay of C4-HSL production using the reporter strain CV026. Purple spots indicate that the diffusible factor was detected by the reporter cells. (**d**) LC-MS analysis of C4-HSL levels of PA1201-derived strains at 12 and 36 h after inoculation in PPM medium. (**e**) LC-MS analysis of PQS in PA1201-derived strains at 12 h and 36 h after inoculation in PPM medium. Values shown are the means ± one SD for three independent experiments. Different letters indicate significant differences between treatments at *P* = 0.05 according to LSD.

To further define the mechanisms underlying the QS signaling systems on PCA biosynthesis, we assayed the production of the three signal molecules 3-oxo-C12-HSL, C4-HSL and PQS in the QS mutant strains of this study. The production of 3-oxo-C12-HSL was assayed using the reporter strain CF11^41^ and strain Δ*lasI* as a negative control. Strain CV026^42^ was used to assay C4-HSL production with Δ*rhlI* as a negative control. C4-HSL, 3-oxo-C12-HSL and PQS were also extracted from the cultures at 12 h and 36 h after inoculation, and their levels were quantified via LC-MS analysis as described by Lépine and Déziel^43^ (Supplementary Figs S3–S5). Deletion of the *mvfR*, *amb* or *pch* clusters, had no significant effect on C4-HSL or 3-oxo-C12-HSL production (Fig. 4a–d). However, the 3-oxo-C12-HSL levels in the Δ*lasR* culture at 12 h and 36 h after inoculation were reduced to approximately 10% and 50% of those in the MSH strain, respectively. Similarly, the C4-HSL levels in the Δ*lasR* culture at 12 h and 36 h after inoculation were approximately 30% and 50% of the MSH strain, respectively. Deletion of *rhlR* had little overall effect on production of 3-oxo-C12-HSL or C4-HSL at 12 h, however, it significantly decreased the C4-HSL levels at 36 h after inoculation (Fig. 4d).

Deletion of *mvfR* abolished PQS production suggesting that MvfR is essential for PQS biosynthesis during growth (Fig. 4e), whereas LasR displayed a growth phase-dependent regulation of PQS production. After 12 h the Δ*lasR* levels were significantly lower than those of MSH; however, after 36 h Δ*lasR* PQS levels were 2.5-fold higher than those of MSH (Fig. 4e). Deletion of *rhlR* had no effect on PQS production 12 h after inoculation, but it did significantly increase PQS production 36 h after inoculation. Deletion of the *amb* or *pch* gene clusters had no significant effect on PQS production during growth (Fig. 4e).

### Transcriptional activities of *phz1* and *phz2* in the MSH strain

**Figure 5:**
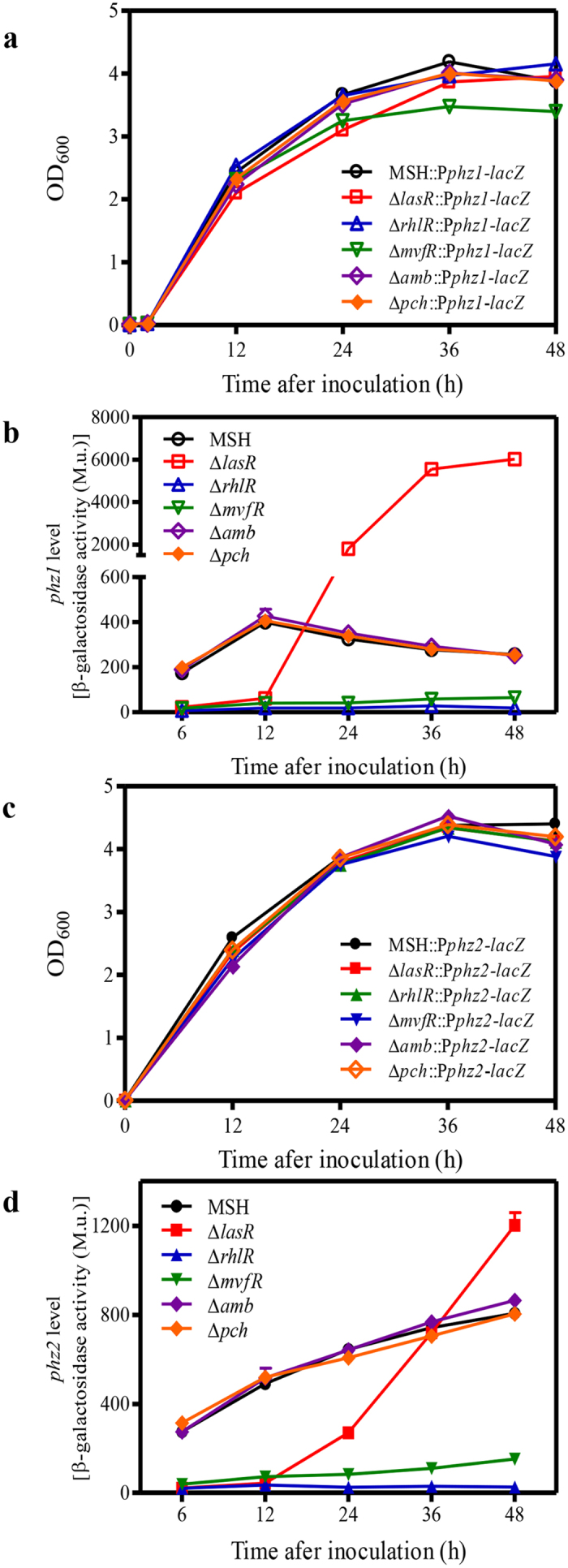
The effects of four QS systems on the expression of *phz* gene clusters in the presence of *phz1* and *phz2*. (**a**) Growth of strains with deletions of individual QS systems and carrying the *phz1* reporter construct. (**b**) The expression of *phz1* in the absence of individual QS systems as indicated by β-galactosidase activities. M. u. indicates Miller unit. (**c**) Growth of strains with deletions of individual QS systems and carrying the *phz2* reporter construct. (**d**) The expression of *phz2* in the absence of individual QS systems as indicated by β-galactosidase activities. The values shown are the means ± one SD from three independent experiments.

PA1201 contains two PCA biosynthetic gene clusters: *phz1* and *phz2* (Fig. 1b). To investigate the relative contributions of *phz1* and *phz2* to PCA biosynthesis in PA1201, we monitored the relative transcriptional activities of *phz1* and *phz2* in the MSH strain. First, total RNAs were extracted from the cell cultures at 12 h and 24 h after inoculation into PPM medium. The transcriptomes were then assayed via RNA-Seq technology. The average expression levels of *phz2* at both 12 h and 24 h were much higher than that of *phz1* (Table 1). During the growth cycle, the average expression of *phz1* decreased from 20 RPKM at 12 h to 7 RPKM at 24 h after inoculation. In contrast, the average expression level of *phz2* increased from 326 RPKM at 12 h to 848 RPKM at 24 h after inoculation (Table 1). We then generated two reporter constructs (P*phz1*-*lacZ* and P*phz2*-*lacZ*) to study the transcriptional activity of *phz1* and *phz2* (as described in Supplementary Fig. S2b). The MSH strains carrying the promoter-*lacZ* fusion constructs displayed similar growth patterns in PPM medium (Fig. 5a,c). The transcriptional activities of *phz1* were significantly lower than those of *phz2* during growth. The maximum level of *phz1* activity was observed at 12 h after inoculation [428 Miller units (M.u.)] (Fig. 5b), whereas the maximum level of *phz2* activity was observed at 48 h after inoculation (807 M.u.) (Fig. 5d). The level of *phz1* activity declined from 428 M.u. at 12 h to 251 M.u. at 48 h (Fig. 5b), whereas the levels of *phz2* activity increased from 516 M.u. at 12 h to 807 M.u. at 48 h (Fig. 5d).

### Effects of four QS systems on the transcriptional activities of *phz1* and *phz2* in the MSH strain

To study the effects of the four QS systems on the expression of the two *phz* clusters, the promoter-*lacZ* fusion constructs (P*phz1-lacZ* and P*phz2-lacZ*) were individually integrated into the chromosomes of the strains MSH, Δ*lasR*, Δ*rhlR*, Δ*mvfR*, Δ*amb*, and Δ*pch*. The resulting strains were grown in PPM medium, and the β-galactosidase activities of the cultures during growth were compared. All of the strains containing the promoter-*lacZ* fusion constructs displayed similar growth patterns in PPM medium (Fig. 5a,c). No significant differences in β-galactosidase activities were observed in strains MSH::P*phz1-lacZ*, Δ*amb*::P*phz1-lacZ*, and Δ*pch*::P*phz1-lacZ* or MSH::P*phz2-lacZ*, Δ*amb*::P*phz2-lacZ*, and Δ*pch*::P*phz2-lacZ*, suggesting that the IQS system had little effect on the expression of *phz1* and *phz2* during growth (Fig. 5b,d). Deletion of the *rhlR* or *mvfR* genes abolished the *phz1* and *phz2* promoter-dependent β-galactosidase activities, respectively (Fig. 5b,d), suggesting that RhlR and MvfR are required for the expression of *phz1* and *phz2*. The regulatory effect of LasR on the expression of *phz1* and *phz2* was dependent on growth phase. LasR induced *phz1* expression at 6 h and 12 h after inoculation and inhibited its expression at 24 h, 36 h and 48 h after inoculation (Fig. 5b). Similarly, LasR induced *phz2* expression at 6 h to 24 h after inoculation, but the inhibitory effect was only observed at 48 h after inoculation (Fig. 5d). Thus, it seems that the inhibitory effect of LasR was greater with *phz1* than with *phz2* during growth.

### Effects of QS systems on *phz1*- and *phz2*-dependent transcriptional activity

**Figure 6:**
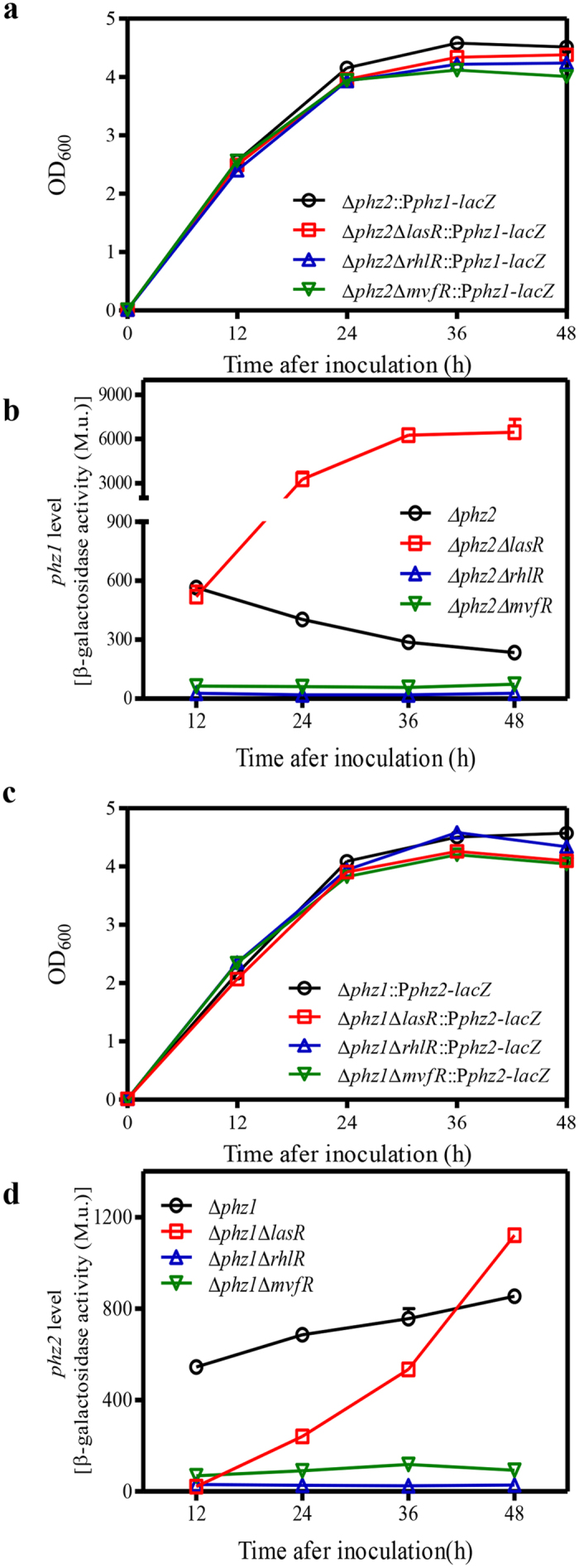
The effects of three QS systems on the expression of one *phz* gene cluster in the absence of the other. (**a**) Growth of strains carrying the *phz1* reporter construct. (**b**) The relative level of *phz1* expression as indicated by β-galactosidase activities in the background of Δ*phz2*. M. u. indicates Miller unit. (**c**) Growth of strains carrying the *phz2* reporter construct. (**d**) The relative level of *phz2* expression as indicated by β-galactosidase activities in the background of Δ*phz1*. The values shown are the means ± one SD from three independent experiments.

Previous results suggested a regulatory feedback loop involving the expressions of two *phz* gene clusters in the strain M18. PCA molecules produced from *phz2* were able to activate the expression of *phz1*^10^. To verify the direct effects of the three QS systems on the expression of *phz1* in the absence of *phz2*, we generated the following strains: Δ*phz2*::P*phz1-lacZ*, Δ*phz2*Δ*lasR*::P*phz1-lacZ*, Δ*phz2*Δ*rhlR*::P*phz1-lacZ*, and Δ*phz2*Δ*mvfR*::P*phz1-lacZ*. These strains displayed similar growth patterns in PPM medium (Fig. 6a). The β-galactosidase activity of these strains was assayed and our results showed that deletion of *rhlR* and *mvfR* abolished *phz1*-dependent transcriptional activity. However, deletion of *lasR* significantly decreased *phz1*-dependent transcriptional activity at 12 h and significantly increased *phz1*-dependent transcriptional activities within 24–48 h after inoculation (Fig. 6b).

To verify the direct effects of the QS systems on the transcriptional activity of *phz2*, we generated the following reporter strains: Δ*phz1*::P*phz2-lacZ*, Δ*phz1*Δ*lasR*::P*phz2-lacZ*, Δ*phz1*Δ*rhlR*::P*phz2-lacZ*, and Δ*phz1*Δ*mvfR*::P*phz2-lacZ*. Deletion of *rhlR* and *mvfR* abolished *phz2*-dependent transcriptional activities. In contrast, deletion of *lasR* led to lower transcriptional activities of *phz2* at 12–36 h and a significantly higher transcriptional activity at 48 h after inoculation (Fig. 6d).

## Discussion

*Pseudomonas aeruginosa* strain PA1201 is a newly identified rhizobacterium that produces high levels of the biopesticide shenqinmycin^39^,^40^. To develop it as an industrial strain for shenqinmycin production, genetic and metabolic engineering of its biosynthetic pathway and regulatory networks is necessary. The QS-dependent regulatory network offers one of the most important engineering targets for improvement of PCA production. This study conducted a global survey and identified the genes for four QS systems and PCA biosynthesis in PA1201. Based on these results, we further compared the effects of four QS systems on bacterial growth, QS signal production, the expression of two *phz* clusters, and PCA biosynthesis in a specialized PPM medium. Our findings establish the LasR, RhlR and MvfR systems as key regulators of PCA biosynthesis in PA1201.

This study makes the following novel contributions to what we know about QS in *P*. *aeruginosa*. First, in most previous studies each QS system was individually investigated. In this study, four QS systems were systematically investigated in the same strain (Figs 5 and 6). Our findings revealed extensive cross-talk among the LasR, RhlR and MvfR systems in PA1201. The roles of RhlR and LasR in regulating PQS production have been investigated for the first time (Fig. 4e). Second, *P*. *aeruginosa* strains usually produce three phenazine derivatives: PCA, PYO and PCN. In most previous studies, the PCA derivative PYO was used as an indicator molecule to evaluate the effects of QS systems on phenazine production. In this study, the effects of QS systems on PCA production were accurately assessed in the strain MSH. Third, all *P*. *aeruginosa* strains contain two nearly identical *phz* clusters and both clusters contribute to PCA production. Li *et al*.^10^ identified a regulatory feedback loop of two *phz* gene clusters expression in M18, which involved a small amount of PCA produced from *phz2* that could function as a signaling molecule to activate *phz1* expression. In most previous QS studies, the feedback regulation of two *phz* clusters was not considered. In this study, the effect of each QS system was individually evaluated in the strain Δ*phz1* or Δ*phz2* (Fig. 6). Our results clearly demonstrated that both MvfR and RhlR systems are required for the induction of *phz1* and *phz2* (Figs 5 and 6). The LasR system had differential effects on the expression of *phz1* and *phz2* during growth (Figs 5 and 6). Thus, our findings present an extended understanding of the regulatory mechanisms of QS systems on the expression of *phz* gene clusters and PCA biosynthesis.

Previous findings showed that the QS circuits in *P. aeruginosa* are organized in a hierarchical manner, and that the *las* system is located at the top of the signaling hierarchy^26^. Consistent with this hierarchy, inactivation of LasR has been reported to severely attenuate QS, the production of quorum-regulated factors, and virulence^44^,^45^,^46^. In this study, LasR negatively regulates the production of 3-oxo-C12-HSL and C4-HSL, and displays a growth phase-dependent regulation of PQS production (Fig. 4). LasR also displayed differential regulation on the expression of *phz1*, *phz2*, and PCA production during growth. The PCA level in the Δ*lasR* culture was significantly lower than that in the MSH culture at early growth stages; however, it was significantly higher in later growth stages (Fig. 2b). This is consistent with recent findings that many clinical *lasR* isolates of *P*. *aeruginosa* can overproduce PYO^18^. The quorum response by *lasR* mutants in slow-growth or stationary-phase conditions is distinct from that in shake culture. *lasR* mutants overproduce PYO under stationary-phase culture conditions^18^. Although Wurtzel *et al*.^47^ previously confirmed the presence of a LasR/RhlR binding site in the promoter region of *phz1* in strain PA14, no *las*/*rhl* box was identified in the promoter region of *phz2*. Therefore, the QS systems might directly regulate *phz* gene expression or affect PCA production indirectly through other factors. The mechanisms underlying the condition-dependent expression of *phz1* and *phz2* are currently under investigation but likely include LasR-dependent regulation.

The newly identified QS signal IQS was shown to positively regulate PYO production in PAO1^24^. However, the present study showed that deletion of the two putative IQS biosynthetic gene clusters had no effect on the expression of *phz1* and *phz2*, or on PCA production in PA1201 (Fig. 2b). There are at least two possibilities for this discrepancy. First, both the *pch* and *amb* gene clusters might not be involved in IQS biosynthesis, or there might be additional pathways for IQS production; second, IQS-dependent regulation of phenazine production could be strain-specific or could depend on the specific growth media used in this study.

**Figure 7:**
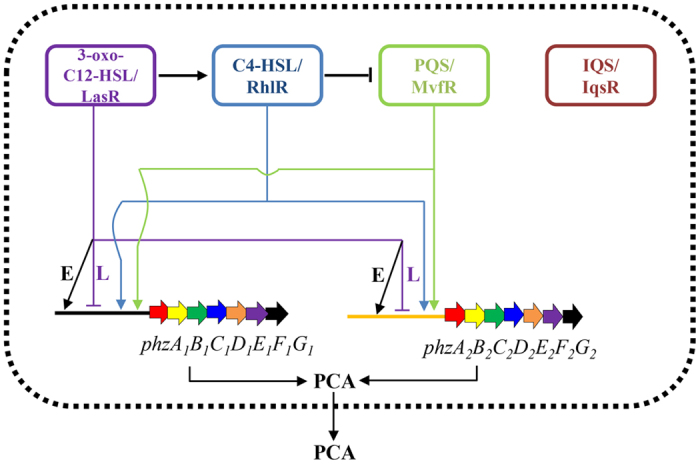
A schematic model for the effect of four QS systems on the expression of the *phz1* and *phz2* gene clusters. The 3-oxo-C12-HSL/LasR system is located at the top of the signaling hierarchy and acts as a global regulator to affect diverse biological activities. It positively regulates C4-HSL production and differentially regulates the expression of both *phz* clusters. At early growth stages, LasR positively regulates the expression of *phz1* and *phz2*, promoting PCA production; at late growth stages, it negatively regulates the expression of *phz1* and *phz2*, repressing PCA production. Both the C4-HSL/RhlR and PQS/MvfR systems positively regulate the expression of *phz1* and *phz2*, promoting PCA production. The C4-HSL/RhlR system slightly represses PQS production in PA1201. The IQS/IqsR system has no effects on other QS systems and does not regulate the expression of *phz1* or *phz2* in PA1201. Arrows indicate positive regulation, and the blunt ends denote negative regulation. The positive regulation of LasR at an early growth stage is indicated by black arrow with “E” for “Early stage”. The negative regulation of LasR at later stage is indicated by purple blunt end with “L” for “Late stage”.

A characteristic feature of *P. aeruginosa* is the presence of two *phz* clusters for PCA production. In this study, we found that both *phz* clusters contributed significantly to PCA production with *phz2* making a greater contribution in PA1201 (Fig. 3). This is consistent with the previous findings in both PA14 and M18 strains^7^,^10^. However, the transcriptional patterns of *phz1* and *phz2* in PA1201 were distinct from previous findings. The transcriptional activities of *phz1* decreased from 12 h to 48 h after inoculation, whereas those of *phz2* increased over time. The transcriptional activities of *phz1* were significantly lower than those of *phz2* during the growth cycle (Figs 5 and 6, Table 1). In previous studies with strain M18, promoter-*lacZ* transcriptional and translational fusions were conducted to monitor *phz1* and *phz2* expression in plasmids pME6522 and pME6015, respectively. In these studies the transcriptional activity of *phz1* was higher than that of *phz2*, whereas the translational activity of *phz1* was lower than that observed with *phz2*^10^. In strain PA14, Recinos *et al*. created *phz* fluorescent reporter constructs with the 500-bp promoter regions upstream of each *phz* cluster fused to *gfp*. These reporter constructs were then integrated into the chromosome. They found that the activities of both *phz* clusters increased over the growth cycle (0–20 hours), and *phz2* was expressed at higher levels than *phz1*^7^. Thus, the relative expression of *phz1* and *phz2* in *P. aeruginosa* appears to be strain-specific. We propose a schematic model for the regulation of *phz1* and *phz2* via the QS systems in PA1201 which is consistent with the results of this study (Fig. 7).

## Methods

### Bacterial strains, plasmids, and culture conditions

The bacterial strains used in this study are listed in Supplementary Table S1. *P. aeruginosa* strain PA1201^39^ was grown in 50 ml of PPM (Pigment-Producing Medium, Tryptone 22 g/L, glucose 20 g/L, KNO_3_ 5 g/L, pH7.5)^48^ or Luria-Bertani (LB) broth in 250 ml flasks in shake culture (200 rpm, 28 °C). *E. coli* strains were cultured in LB medium at 37 °C. When required, antibiotics were added to the medium at the following final concentrations: spectinomycin (50 μg ml^−1^), tetracycline (100 μg ml^−1^ for *P. aeruginosa* strains and 10 μg ml^−1^ for *E.coli* strains), and gentamycin (100 μg ml^−1^).

### Generation of *in-frame* deletion mutants in PA1201

The methods for *in-frame* gene deletion followed the general procedure shown in Supplementary Fig. S2. Briefly, the downstream and upstream regions of the target gene to be deleted were combined by using overlap extension PCR. The fusion products were further cloned into the suicide vector pEX18Gm^49^ carrying the sucrose-sensitive *sacB* gene. The resulting chimeric plasmid was then integrated within the target sequence via homologous recombination, and plasmid sequences were then removed by a second single-crossover event, resulting in allelic exchange^50^. The plasmids and primers used in this study are listed in Supplementary Tables S1 and S2. The markerless mutants generated were verified by colony PCR and subsequent DNA sequencing.

### Extraction and quantification of phenazine-1-carboxylic acid

For PCA extraction, 180 μl of fermentation culture was mixed with 20 μl of 6 M HCl and then extracted with 540 μl chloroform as previously described^40^. A 3 μl aliquot of condensed PCA extract was then taken for HPLC analysis (Agilent Technologies 1260 Infinity) under the following conditions: C18 reversed-phase column (5 μm, 4.6 × 150 mm) eluted with acetonitrile-5 mM ammonium acetate (60:40, v/v). PCA production was quantified using peak area (A) in the HPLC elute according to the following formula: PCA (mg/L) = 0.0146A-0.341. This was derived from a dose–peak area plot using purified PCA with a correlation coefficient (R^2^) of 0.999.

### Detection and bioassay analysis of C4-HSL and 3-oxo-C12-HSL production

The production of C4-HSL and 3-oxo-C12-HSL in PA1201 and derived mutant strains was detected and analyzed using a diffusion plate as previously described^41^,^42^. The previously constructed strain *A. tumefaciens* CF11 was used to detect 3-oxo-C12-HSL production^41^. *C. violaceum* strain CV026 was used to detect short chain C4-HSL production^42^. Blue or purple spots indicated that the diffusible QS signals were detected by the reporter cells. The production of C4-HSL and 3-oxo-C12-HSL is proportional to the diffusion distance from the last purple or blue spot to the origin of PA1201 strains.

### Extraction and quantification of C4-HSL, 3-oxo-C12-HSL, and PQS by LC-MS

For extraction of QS signaling molecules, 270 μl of culture fluid was collected and adjusted to pH = 4.0 by the addition of 6 M HCl. This was then extracted with an equal volume of ethyl acetate. Subsequently 100 μl of ethyl acetate extract was collected for evaporation at 40 °C, and the resulting residue was finally dissolved in 500 μl of methanol. A ten-microliter aliquot of this sample was then injected into an ultra-performance liquid chromatography column which was coupled with mass spectrometry (Agilent UPLC1290-TOF-MS6230) under the following conditions: Agilent Zorbax XDB C18 reverse-phase (5 μm, 4.6 × 150 mm) eluted with gradient ACN with 0.5% acetic acid and H_2_O with 0.5% acetic acid at 0.4 ml/min. The MS analysis was performed under positive mode with a scanning range of m/z = 100–1700. The specific pseudo molecular ion (M+H)^+^ or (M+Na)^+^ of 3-oxo-C12-HSL, C4-HSL, and PQS were extracted at 320.1832, 194.0788, and 260.1645, respectively. The retention times of 3-oxo-C12-HSL, C4-HSL, and PQS were 13.68 min, 7.67 min, and 12.60 min, respectively. The commercially available C4-HSL (Cayman Chemical, Michigan, USA), 3-oxo-C12-HSL and PQS (Sigma-Aldrich) were also assayed using LC-MS and the standard curves derived from a dose–peak area plot were established (Supplementary Figs S3–S5). The concentration of QS molecules was quantified with a peak area (A) of the specific extracted ion chromatogram (EIC) in the total ion chromatogram (TIC) according to the following formula: 3-oxo-C12-HSL (μM) = 6 × 10^−7^A. This was derived from a dose–peak area plot using standard 3-oxo-C12-HSL with a correlation coefficient (R^2^) of 0.979. The C4-HSL (μM) = 2 × 10^−6^A with a R^2^ of 0.995, and PQS (μM) = 3 × 10^−6^A + 0.4728 with a R^2^ of 0.966.

### RNA Sequencing

RNA-Seq analysis was conducted at BGI (http://www.genomics.cn). In brief, total RNA was extracted from 1.5 ml of cell cultures at 12 h and 24 h after inoculation using the RNeasy Miniprep Kit. After DNA contamination was removed with RNase-Free DNase Set (Qiagen), the 1 μg sample of total RNA was treated with Ribo-Zero^TM^ Magnetic Gold Kit (Epicenter) to remove rRNA. Fragmentation buffer was then added to break mRNA into short fragments, and random hexamer-primers were subsequently used to synthesize first-strand cDNA. After removal of dNTPs, second-strand cDNA was then synthesized with buffer, dATPs, dGTPs, dCTPs, dUTPs, RNase H and DNA polymerase I. Short fragments were subsequently purified with the QiaQuick^®^PCR extraction kit and poly(A) and sequencing adapters were added. The UNG enzyme was then used to degrade the second-strand cDNA, and the product was purified by MiniElute PCR Purification Kit before PCR amplification. The qualified library was amplified on cBot to generate the cluster on the flowcell (TruSeq PE Cluster Kit V3–cBot–HS, Illumina). The amplified flowcell was paired-end sequenced on the HiSeq 2000 System. For the two samples at different points, a total of 214062560bp and 1174736520 bp, respectively, were sequenced. The reads were aligned to the genome sequences by the program SOAPaligner/soap2. The gene expression level was calculated by using RPKM method^51^ (Reads per kilobase transcriptome per million mapped reads), and the formula is as follows: RPKM = 10^6^C/(NL/10^3^). Given to be the expression of gene A, C to be the number of reads that are uniquely aligned to gene A, N to be the total number of reads that are uniquely aligned to all genes, and L to be the number of bases in the gene. The original data are available in NCBI Sequence Read Archive (Accession SRP074264).

### Construction of the promoter-*lacZ* fusion reporter strains and expression analysis of *phz1* and *phz2*

The reporter constructs used to monitor the expression of *phz1* or *phz2* were generated with a previously described method^52^. Briefly, the 500-bp promoter regions and 30-bp coding sequences of *phzA1* or *phzA2* were individually cloned into the vector mini-CTX-*lacZ*. The resulting constructs, mini-CTX-P*phz1*-lacZ and mini-CTX-P*phz2*-lacZ, were integrated into the chromosomes of PA1201-derived strains. The β-galactosidase activity was measured as previously described^53^.

### Statistical analysis

Analysis of variance for experimental datasets was performed using the JMP software version 5.0 (SAS Institute Inc., Cary, NC). Significant effects of treatment were determined by the *F* value (*P* = 0.05). When a significant *F* test was obtained, separation of means was accomplished via Fisher’s protected LSD (least significant difference) at a significance level of *P* = 0.05.

## Additional Information

**How to cite this article**: Sun, S. *et al*. Quorum-sensing systems differentially regulate the production of phenazine-1-carboxylic acid in the rhizobacterium *Pseudomonas aeruginosa* PA1201. *Sci. Rep*. **6**, 30352; doi: 10.1038/srep30352 (2016).

## Supplementary Material

Supplementary Information

## Figures and Tables

**Table 1:** The relative expression of *phz* clusters as revealed by RNA-Seq analysis.

Genes	Expression level (RPKM)	
	24 h	24 h
*phzA1*	45	16
*phzB1*	14	6
*phzC1*	24	4
*phzD1*	15	12
*phzE1*	9	4
*phzF1*	5	1
*phzG1*	26	8
**Mean**	**20**	**7**
*phzA2*	168	639
*phzB2*	1207	3351
*phzC2*	168	409
*phzD2*	192	334
*phzE2*	ND	ND
*phzF2*	59	105
*phzG2*	163	249
**Mean**	**326**	**848**

RPKM (Reads Per kb per Million reads) was used to calculate the gene expression level. ND: no data.

## References

[b1] Mazzola M., Cook R. J., Thomashow L. S., Weller D. M. & Pierson L. S. Contribution of phenazine antibiotic biosynthesis to the ecological competence of fluorescent pseudomonads in soil habitats. Appl. Environ. Microbiol. 58, 2616–2624 (1992). 1514808 10.1128/aem.58.8.2616-2624.1992 PMC195829

[b2] Pierson L. S. III. & Pierson E. A. Phenazine antibiotic production by the biological control bacterium *Pseudomonas aureofaciens*: role in ecology and disease suppression. FEMS Microbol. Lett. 136, 101–108 (2006).

[b3] Mavrodi D. V., Blankenfeldt W. & Thomashow L. S. Phenazine compounds in fluorescent *Pseudomonas* spp. biosynthesis and regulation. Annu. Rev. Phytopathol 44, 417–445 (2006). 16719720 10.1146/annurev.phyto.44.013106.145710

[b4] De Vleesschauwer D., Djavaheri M., Bakker P. A. & Höfte M. *Pseudomonas fluorescens* WCS374r-induced systemic resistance in rice against *Magnaporthe oryzae* is based on pseudobactin-mediated priming for a salicylic acid-repressible multifaceted defense response. Plant Physiol. 148 **(4)**, 1996–2012 (2008). 18945932 10.1104/pp.108.127878 PMC2593667

[b5] Dietrich L. E., Price-Whelan A., Petersen A., Whiteley M. & Newman D. K. The phenazine pyocyanin is a terminal signalling factor in the quorum sensing network of *Pseudomonas aeruginosa*. Mol Microbiol. 61 **(5)**, 1308–1321 (2006). 16879411 10.1111/j.1365-2958.2006.05306.x

[b6] Caldwell C. C. . *Pseudomonas aeruginosa* exotoxin pyocyanin causes cystic fibrosis airway pathogenesis. Am. J. Pathol. 175, 2473–2488 (2009). 19893030 10.2353/ajpath.2009.090166 PMC2789600

[b7] Recinos D. A. . Redundant phenazine operons in *Pseudomonas aeruginosa* exhibit environment-dependent expression and differential roles in pathogenicity. Proc. Natl. Acad. Sci. USA 109, 19420–19425 (2012). 23129634 10.1073/pnas.1213901109 PMC3511076

[b8] Thomashow L. In Microbial Phenazines: Biosynthesis, Agriculture and Health (eds Chincholkar S. & Thomashow L.) Ch. 10, 199–216 (Springer Heidelberg, 2013).

[b9] Mentel M. . Of two make one: the biosynthesis of phenazines. ChemBioChem. 10, 2295–2304 (2009). 19658148 10.1002/cbic.200900323

[b10] Li Y. . Regulatory feedback loop of two phz gene clusters through 5’-untranslated regions in *Pseudomonas* sp. M18. PLos One 6 **(4)**, e19413 (2011). 21559370 10.1371/journal.pone.0019413 PMC3084852

[b11] Cui Q. . Cross-Regulation between the *phz1* and *phz2* operons maintain a balanced level of phenazine biosynthesis in *Pseudomonas aeruginosa* PAO1. PLos One 11(1), e0144447 (2016). 26735915 10.1371/journal.pone.0144447 PMC4703396

[b12] Chin-A-Woeng T. F., Thomas-Oates J. E., Lugtenberg B. J. & Bloemberg G. V. Introduction of the *phzH* gene of *Pseudomonas chlororaphis* PCL1391 extends the range of biocontrol ability of phenazine-1-carboxylic acid-producing *Pseudomonas* spp. strains. Mol. Plant Microbe Interact. 14, 1006–1015 (2001). 11497461 10.1094/MPMI.2001.14.8.1006

[b13] Delaney S. M., Mavrodi D. V., Bonsall R. F. & Thomashow L. S. *phzO*, a gene for biosynthesis of 2-hydroxylated phenazine compounds in *Pseudomonas aureofaciens* 30–84. J. Bacteriol. 183, 318–327 (2001). 11114932 10.1128/JB.183.1.318-327.2001 PMC94881

[b14] Mavrodi D. V. . Functional analysis of genes for biosynthesis of pyocyanin and phenazine-1-carboxamide from *Pseudomonas aeruginosa* PAO1. J. Bacteriol. 183, 6454–6465 (2001). 11591691 10.1128/JB.183.21.6454-6465.2001 PMC100142

[b15] Greenhagen B. T. . Crystal structure of the pyocyanin biosynthetic protein PhzS. Biochemistry 47, 5281–5289 (2008). 18416536 10.1021/bi702480t

[b16] Huang J. . Temperature-dependent expression of *phzM* and its regulatory genes *lasI* and *ptsP* in rhizosphere isolate *Pseudomonas* sp. strain M18. Appl. Environ. Microbiol. 75, 6568–6580 (2009). 19717631 10.1128/AEM.01148-09 PMC2765144

[b17] Sakhtah H., Price-Whelan A. & Dietrich L. E. In Microbial Phenazines: Biosynthesis, Agriculture and Health (eds Chincholkar S. & Thomashow L.) Ch. 2, 19–42 (Springer Heidelberg, 2013).

[b18] Cabeen M. T. Stationary phase-specific virulence factor overproduction by a *lasR* mutant of *Pseudomonas aeruginosa*. PLos One. 9(2), e88743 (2014). 24533146 10.1371/journal.pone.0088743 PMC3923063

[b19] Wanger V. E., Bushnell D., Passador L., Brooks A. I. & Iglewski B. H. Microarray analysis of *Pseudomonas aeruginosa* quorum-sensing regulons: effects of growth phase and environment. J. Bacteriol. 185(7), 2080–2095 (2003). 12644477 10.1128/JB.185.7.2080-2095.2003 PMC151498

[b20] Williams P. & Camara M. Quorum sensing and environmental adaptation in *Pseudomonas aeruginosa*: a tale of regulatory networks and multifunctional signal molecules. Curr Opin Microbiol. 12(2), 182–191 (2009). 19249239 10.1016/j.mib.2009.01.005

[b21] Sonnleitner E. & Haas D. Small RNAs as regulators of primary and secondary metabolism in *Pseudomonas* species. Appl Microbiol Biotechnol. 91(1), 63–79 (2011). 21607656 10.1007/s00253-011-3332-1

[b22] Jimenez P. N. . The multiple signaling systems regulating virulence in *Pseudomonas aeruginosa*. Microbiol. Mol. Biol. Rev. 76, 46–65 (2012). 22390972 10.1128/MMBR.05007-11 PMC3294424

[b23] Balasubramanian D., Schneper L., Kumari H. & Mathee K. A dynamic and intricate regulatory network determines *Pseudomonas aeruginosa* virulence. Nucleic Acids Res. 41, 1–20 (2013). 23143271 10.1093/nar/gks1039 PMC3592444

[b24] Lee J. . A cell-cell communication signal integrates quorum sensing and stress response. Nat. Chem. Biol. 9, 339–343 (2013). 23542643 10.1038/nchembio.1225

[b25] Lee J. & Zhang L. The hierarchy quorum sensing network in *Pseudomonas aeruginosa*. *Protein* Cell 6, 26–41 (2015). 10.1007/s13238-014-0100-x PMC4286720 25249263

[b26] Fuqua W. C., Winans S. C. & Greenberg E. P. Quorum sensing in bacteria: the LuxR-LuxI family of cell density-responsive transcriptional regulators. J. Bacteriol. 176, 269–275 (1994). 8288518 10.1128/jb.176.2.269-275.1994 PMC205046

[b27] Pearson J. P., Passador L., Iglewski B. H. & Greenberg E., P. A second N-acylhomoserine lactone signal produced by *Pseudomonas aeruginosa*. Proc. Natl. Acad. Sci. USA 92, 1490–1494 (1995). 7878006 10.1073/pnas.92.5.1490 PMC42545

[b28] Fuqua C., Winans S. C. & Greenberg E. P. Census and consensus in bacterial ecosystems: the LuxR-LuxI family of quorum-sensing transcriptional regulators. Annu. Rev. Microbiol. 50, 727–751 (1996). 8905097 10.1146/annurev.micro.50.1.727

[b29] Bredenbruch F. . Biosynthetic pathway of *Pseudomonas aeruginosa* 4-hydroxy-2-alkylquinolines. J. Bacteriol. 187, 3630–3635 (2005). 15901684 10.1128/JB.187.11.3630-3635.2005 PMC1112037

[b30] Diggle S. P., Cornelis P., Williams P. & Cámara M. 4-quinolone signalling in *Pseudomonas aeruginosa*: old molecules, new perspectives. Int. J. Med. Microbiol. 296, 83–91 (2006). 16483840 10.1016/j.ijmm.2006.01.038

[b31] Ye L., Cornelis P., Guillemyn K., Ballet S. & Hammerich O. Structure revision of N-mercapto-4-formylcarbostyril produced by *Pseudomonas fluorescens* G308 to 2-(2-hydroxyphenyl) thiazole-4-carbaldehyde [aeruginaldehyde]. Nat Prod Commun. 9(6), 789–794 (2014). 25115080

[b32] Murcia R. N. . The *Pseudomonas aeruginosa* antimetabolite L -2-amino-4-methoxy-*trans*-3-butenoic acid (AMB) is made from glutamate and two alanine residues via a thiotemplate-linked tripeptide precursor. Front Microbiol. 6, 170 (2015). 25814981 10.3389/fmicb.2015.00170 PMC4357302

[b33] Dekimpe V. & Déziel E. Revisiting the quorum-sensing hierarchy in *Pseudomonas aeruginosa:* the transcriptional regulator RhlR regulates LasR-specific factors. Microbiology 155, 712–723 (2009). 19246742 10.1099/mic.0.022764-0

[b34] Lu J. . The distinct quorum sensing hierarchy of *las* and *rhl* in *Pseudomonas* sp. M18. Curr. Microbiol. 59, 621–627 (2009). 19727948 10.1007/s00284-009-9483-y

[b35] Hu H. B., Xu Y. Q., Chen F., Zhang X. H. & Hur B. K. Isolation and characterization of a new fluorescent *Pseudomonas* strain that produces both phenazine 1-carboxylic acid and pyoluteorin. J. Microbiol. Biotechnol. 15, 86–90 (2005).

[b36] Li Y. . Enhancement of phenazine-1-carboxylic acid production using batch and fed-batch culture of gacA inactivated *Pseudomonas* sp. M18G. Bioresour. Technol. 101, 3649–3656 (2010). 20097558 10.1016/j.biortech.2009.12.120

[b37] Du X. . Phenazine-1-carboxylic acid production in a chromosomally non-scar triple-deleted mutant *Pseudomonas aeruginosa* using statistical experimental designs to optimize yield. Appl. Microbiol. Biotechnol. 97, 7767–7778 (2013). 23636695 10.1007/s00253-013-4921-y

[b38] Xu Y. In Microbial Phenazines: Biosynthesis, Agriculture and Health (eds Chincholkar S. & Thomashow L.) Ch. 9, 177–198 (Springer Heidelberg, 2013).

[b39] Zhou L. . Biotechnological potential of a rhizosphere *Pseudomonas aeruginosa* strain producing phenazine-1-carboxylic acid and phenazine-1-carboxamide. World J. Microbiol. Biotechnol. 32(3), 50 (2016). 26873561 10.1007/s11274-015-1987-y

[b40] Jin K. . Engineering the central biosynthetic and secondary metabolic pathways of *Pseudomonas aeruginosa* strain PA1201 to improve phenazine-1-carboxylic acid production. Metab Eng. 32, 30–38 (2015). 26369437 10.1016/j.ymben.2015.09.003

[b41] Zhang H. B., Wang L. H. & Zhang L. H. Detection and analysis of quorum-quenching enzymes against acyl homoserine lactone quorum-sensing signals. Curr Protoc Microbiol Ch1, Unit 1C. 3 (2007). 10.1002/9780471729259.mc01c03s05 18770600

[b42] McClean K. H. . Quorum sensing and *Chromobacterium violaceum*: exploitation of violacein production and inhibition for the detection of N-acylhomoserine lactones. Microbiology. 143, 3703–3711 (1997). 9421896 10.1099/00221287-143-12-3703

[b43] Lépine F. & Déziel E. Liquid chromatography/mass spectrometry for the detection and quantification of N-acyl-L-homoserine lactones and 4-hydroxy-2-alkylquinolines. Methods Mol. Biol. 692, 61–69 (2011). 21031304 10.1007/978-1-60761-971-0_5

[b44] Schuster M., Lostroh C. P., Ogi T. & Greenberg E. P. Identification, timing, and signal specificity of *Pseudomonas aeruginosa* quorum-controlled genes: a transcriptome analysis. J Bacteriol 185(7), 2066–2079 (2003). 12644476 10.1128/JB.185.7.2066-2079.2003 PMC151497

[b45] Smith R. S. & Iglewski B. H. *P. aeruginosa* quorum-sensing systems and virulence. Curr Opin Microbiol 6(1), 56–60 (2003). 12615220 10.1016/s1369-5274(03)00008-0

[b46] D’Argenio D. A. . Growth phenotypes of *Pseudomonas aeruginosa lasR* mutants adapted to the airways of cystic fibrosis patients. Mol Microbiol 64(2), 512–533 (2007). 17493132 10.1111/j.1365-2958.2007.05678.x PMC2742308

[b47] Wurtzel O. . The single-nucleotide resolution transcriptome of *Pseudomonas aeruginosa* grown in body temperature. PLoS Pathog. 8(9), e1002945 (2012). 23028334 10.1371/journal.ppat.1002945 PMC3460626

[b48] Shtark O. Y., Shaposhnikov A. I. & Kravchenko L. V. The production of antifungal metabolites by *Pseudomonas chlororaphis* grown on different nutrient sources. Microbiology 72(5), 574–578 (2003). 14679903

[b49] Hoang T. T., Karkhoff-Schweizer R. R., Kutchma A. J. & Schweizer H. P. A broad-host-range Flp-FRT recombination system for site-specific excision of chromosomally-located DNA sequences: application for isolation of unmarked Pseudomonas aeruginosa mutants. Gene 212, 77–86 (1998). 9661666 10.1016/s0378-1119(98)00130-9

[b50] He Y. W. . Genome scale analysis of diffusible signal factor regulon in *Xanthomonas campestris pv. campestris*: identification of novel cell-cell communication-dependent genes and functions. Mol. Microbiol. 59, 610–622 (2006). 16390454 10.1111/j.1365-2958.2005.04961.x

[b51] Mortazavi A., Williams B. A., McCue K., Schaeffer L. & Wold B. Mapping and quantifying mammalian transcriptomes by RNA-Seq. Nat Methods. 5(7), 621–628 (2008). 18516045 10.1038/nmeth.1226 PMC13303166

[b52] Becher A. & Schweizer H. P. Integration-proficient *Pseudomonas aeruginosa* vectors for isolation of single-copy chromosomal lacZ and lux gene fusions. Biotechniques 29, 948–950 (2000). 11084852 10.2144/00295bm04

[b53] Sambrook J. & Russell D. W. In Molecular cloning: a laboratory manual 3rd ed. (Cold Spring Laboratory Press, 2001).

