# Supplementary material for: Quorum sensing systems differentially regulate the production of phenazine-1-carboxylic acid in the rhizobacterium Pseudomonas aeruginosa PA1201
Source: Sci Rep. 2016 Jul 26;6:30352. doi: 10.1038/srep30352 (PMC4960564; doi:10.1038/srep30352)
Supplement: Supplementary Information [file srep30352-s1.pdf]

Supplementary Material

Quorum sensing systems differentially regulate the production of phenazine-1-carboxylic acid in the rhizobacterium *Pseudomonas aeruginosa* PA1201

Authors: Shuang Sun[1], Lian Zhou[1], Kaiming Jin[1], Haixia Jiang[1], Ya-Wen He[1*]

[1]State Key Laboratory of Microbial Metabolism, School of Life Sciences & Biotechnology, Shanghai Jiao Tong University, Shanghai 200240, China

Contents

Supplementary Figure S1

Supplementary Figure S2

Supplementary Figure S3

Supplementary Figure S4

Supplementary Figure S5

Supplementary TableS1

Supplementary TableS2

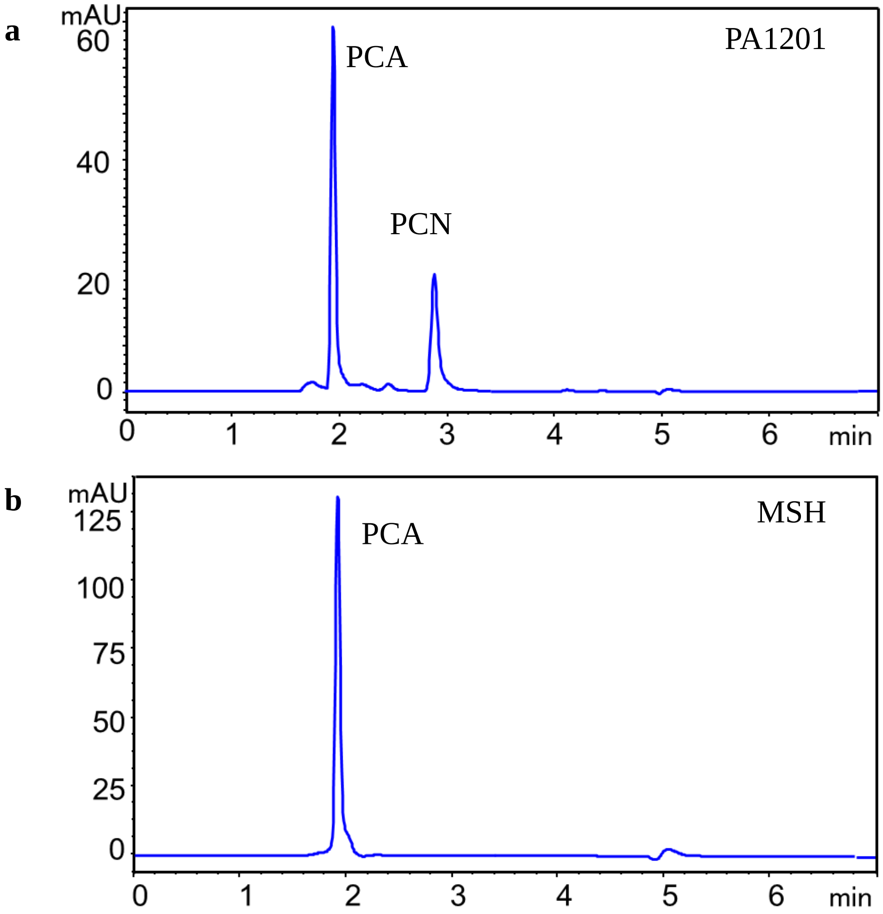

Supplementary Figure S1 HPLC analysis of strain PA1201 and strain MSH with mutations in *phzM*, *phzS*, and *phzH*.

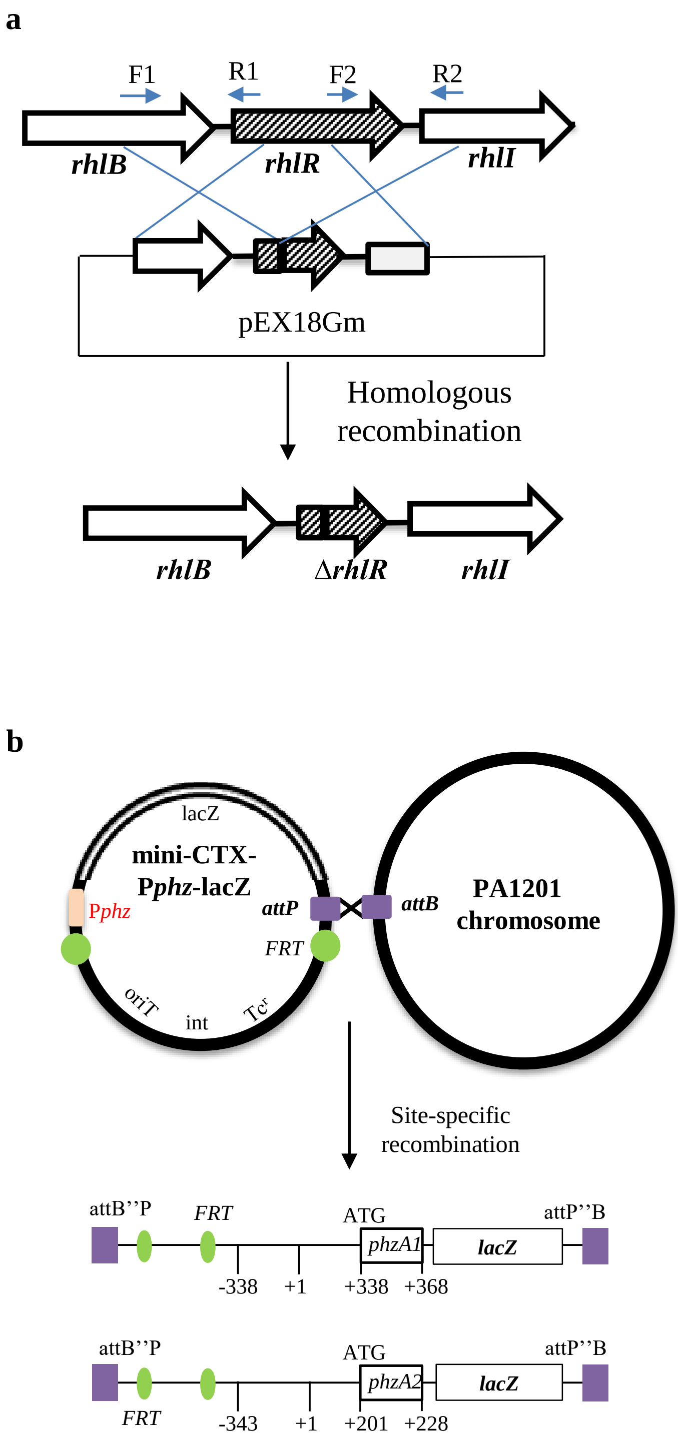

Supplementary Figure S2 The schematic strategies for (a) *in-frame* gene deletion and (b) construction of a single copy reporter for the gene cluster *phz1* or *phz2*.

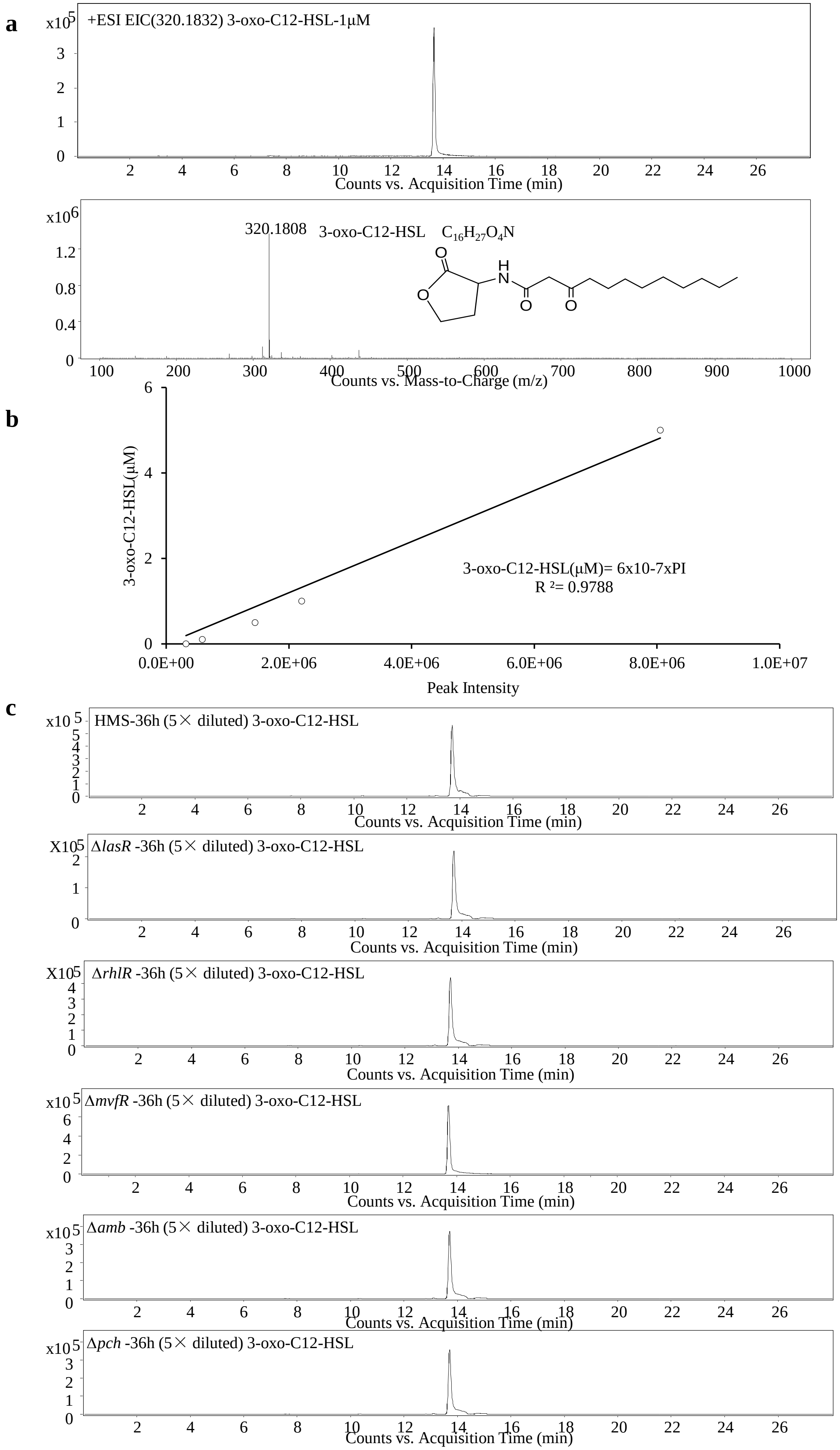

Supplementary Figure S3 LC-MS analysis of 3-oxo-C12-HSL production in various deletion strains. (a) LC-MS analysis of the commercially available 3-oxo-C12-HSL samples at different concentrations. (b) The standard curve of peak intensity for the specific extracted ion chromatogram (EIC) and 3-oxo-C12-HSL concentrations. (c) EIC chromatograms of extracted 3-oxo-C12-HSL samples.

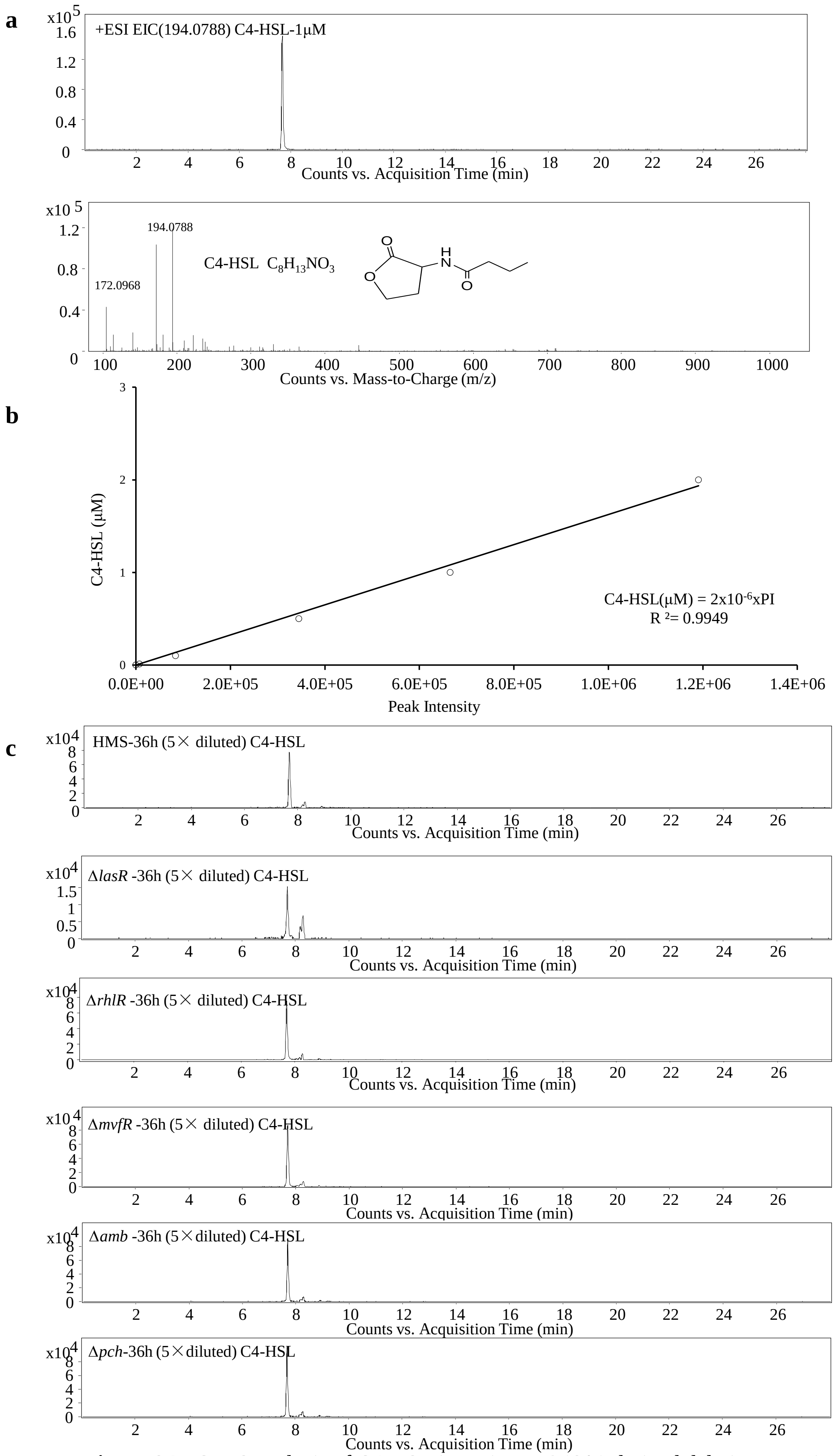

Supplementary Figure S4 LC-MS analysis of C4-HSL in various PA1201-derived deletion mutation strains. (a) LC-MS analysis of the commercially available C4-HSL. (b) The standard curve of peak intensity for the specific extracted ion chromatogram (EIC) and C4-HSL concentrations. (c) EIC chromatograms of extracted C4-HSL samples

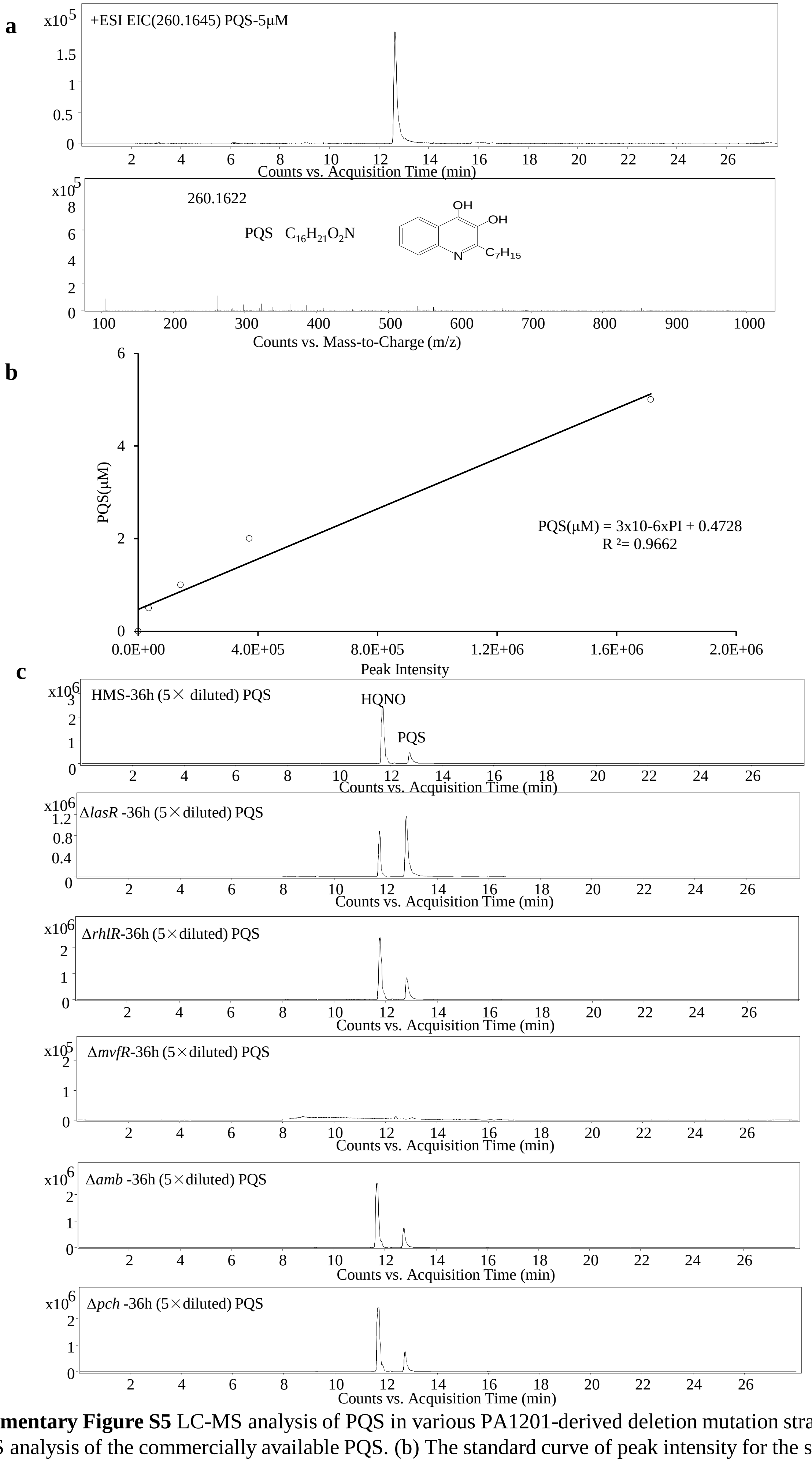

Supplementary Figure S5 LC-MS analysis of PQS in various PA1201-derived deletion mutation strains. (a) LC-MS analysis of the commercially available PQS. (b) The standard curve of peak intensity for the specific extracted ion chromatogram (EIC) and PQS concentrations. (c) EIC chromatograms of extracted PQS samples.

Supplementary Table S1 The strains and plasmids used in this study

Strains	Relevant characteristics	Reference or source
P. aeruginosa strains		
PA1201	*P. aeruginosa* wild-type strain, Spe^R	39
MSH	PA1201Δ*phzM*Δ*phzS*Δ*phzH* The *phzM, phzS* and *phzH* in-frame deletion mutant, Spe^R	40
MSHΔ*lasR*	*lasR* in-frame deletion mutant based on MSH, Spe^R	This study
MSHΔ*rhlR*	*rhlR* in-frame deletion mutant based on MSH, Spe^R	This study
MSHΔ*mvfR*	*mvfR* in-frame deletion mutant based on MSH, Spe^R	This study
MSHΔ*amb*	*ambBCDE* in-frame deletion mutant based on MSH, Spe^R	This study
MSHΔ*pch*	*pchABCDREFG* in-frame deletion mutant based on MSH, Spe^R	This study
MSHΔ*phz1*	$phzA_1B_1C_1D_1E_1F_1$ G_1in-frame deletion mutant based on MSH, Spe^R	40
MSHΔ*phz1*Δ*lasR*	*lasR* in-frame deletion mutant based on MSHΔ*phz1*, Spe^R	This study
MSHΔ*phz1*Δ*rhlR*	*rhlR* in-frame deletion mutant based on MSHΔ*phz1*, Spe^R	This study
MSHΔ*phz1*Δ*mvfR*	*mvfR* in-frame deletion mutant based on MSHΔ*phz1*, Spe^R	This study
MSHΔ*phz2*	$phzA_2B_2C_2D_2E_2F_2$ G_2in-frame deletion mutant based on MSH, Spe^R	40
MSHΔ*phz2*Δ*lasR*	*lasR* in-frame deletion mutant based on MSHΔ*phz2*, Spe^R	This study
MSHΔ*phz2*Δ*rhlR*	*rhlR* in-frame deletion mutant based on MSHΔ*phz2*, Spe^R	This study
MSHΔ*phz2*Δ*mvfR*	*mvfR* in-frame deletion mutant based on MSHΔ*phz2*, Spe^R	This study
MSH::P*phz1*-lacZ	Single copy insertion of P*phz1*-lacZ in the MSH chromosomal attB site	This study
MSHΔ*lasR*::P*phz1*-lacZ	Single copy insertion of P*phz1*-lacZ in the MSHΔ*lasR* chromosomal attB site	This study
MSHΔ*rhlR*::P*phz1*-lacZ	Single copy insertion of P*phz1*-lacZ in the MSHΔ*rhlR* chromosomal attB site	This study
MSHΔ*mvfR*::P*phz1*-lacZ	Single copy insertion of P*phz1*-lacZ in the MSHΔ*mvfR* chromosomal attB site	This study
MSHΔ*amb*::P*phz1*-lacZ	Single copy insertion of P*phz1*-lacZ in the MSHΔ*amb* chromosomal attB site	This study
MSHΔ*pch*::P*phz1*-lacZ	Single copy insertion of P*phz1*-lacZ in the MSHΔ*pch* chromosomal attB site	This study
MSH::P*phz2*-lacZ	Single copy insertion of P*phz2*-lacZ in the MSH chromosomal attB site	This study
MSHΔ*lasR*::P*phz2*-lacZ	Single copy insertion of P*phz2*-lacZ in the MSHΔ*lasR* chromosomal attB site	This study
MSHΔ*rhlR*::P*phz2*-lacZ	Single copy insertion of P*phz2*-lacZ in the MSHΔ*rhlR* chromosomal attB site	This study
MSHΔ*mvfR*::P*phz2*-lacZ	Single copy insertion of P*phz2*-lacZ in the MSHΔ*mvfR* chromosomal attB site	This study
MSHΔ*amb*::P*phz2*-lacZ	Single copy insertion of P*phz2*-lacZ in the MSHΔ*amb* chromosomal attB site	This study
MSHΔ*pch*::P*phz2*-lacZ	Single copy insertion of P*phz2*-lacZ in the MSHΔ*pch* chromosomal attB site	This study
MSHΔ*phz2*::P*phz1*-lacZ	Single copy insertion of P*phz1*-lacZ in the MSHΔ*phz2* chromosomal attB site	This study
MSHΔ*phz2*Δ*lasR*::P*phz1*-lacZ	Single copy insertion of P*phz1*-lacZ in the MSHΔ*phz2*Δ*lasR* chromosomal attB site	This study
MSHΔ*phz2*Δ*rhlR*::P*phz1*-lacZ	Single copy insertion of P*phz*1-lacZ in the MSHΔ*phz2*Δ*rhlR* chromosomal attB site	This study
MSHΔ*phz2*Δ*mvfR*::P*phz1*-lacZ	Single copy insertion of P*phz1*-lacZ in the MSHΔ*phz2*Δ*mvfR* chromosomal attB site	This study
MSHΔ*phz1*::P*phz2*-lacZ	Single copy insertion of P*phz2*-lacZ in the MSHΔ*phz1* chromosomal attB site	This study
MSHΔ*phz1*Δ*lasR*::P*phz2*-lacZ	Single copy insertion of P*phz2*-lacZin the MSHΔ*phz1*Δ*lasR* chromosomal attB site	This study
MSHΔ*phz1*Δ*rhlR*::P*phz2*-lacZ	Single copy insertion of P*phz2*-lacZ in the MSHΔ*phz1*Δ*rhlR* chromosomal attB site	This study
MSHΔ*phz1*Δ*mvfR*::P*phz*2-lacZ	Single copy insertion of P*phz2*-lacZ in the MSHΔ*phz1*Δ*mvfR* chromosomal attB site	This study

Supplementary Table S1 The strains and plasmids used in this study

Strains	Relevant characteristics	Reference or source
Reporter strains		
C. violaceum CV026	Double mini-Tn5 mutant derived from *C. violaceum* ATCC 31532, AHL biosensor	42
A.Tumefaciens CF11	Harbors two plasmids expressing TraR and the reporter gene lacZ under the tra reporter.respectively	41
E. coli strains		
DH5α	*E. coli* F– Φ80lacZΔM15 Δ(lacZYA-argF) U169 recA1 endA1 hsdR17 (rK–, mK+) phoA supE44 λ– thi-1 gyrA96 relA1	Lab collection
pRK2013	A conjugative plasmid,Kan^R	Lab collection
S17-1λpir	TpR SmR recA, thi, pro, hsdR-M+RP4: 2-Tc:Mu: Kan Tn7 λpir	Lab collection
Plasmids		
pEX18Gm	Gm^R; $oriT^+$ $sacB^+$, gene replacement vector with MCS This study from pUC18	49
mini-CTX-lacZ	Tet^R; integration-proficient vector for chromosomal insertion at the attB site	52
pEX18Gm-Δ*lasR*	pEX18Gm with the fusion product of the *lasR* flanking fragments for in-frame deletion of *lasR*, Gm^R	This study
pEX18Gm-Δ*rhlR*	pEX18Gm with the fusion product of the *rhlR* flanking fragments for in-frame deletion of *rhlR*, Gm^R	This study
pEX18Gm-Δ*mvfR*	pEX18Gm with the fusion product of the *mvfR* flanking fragments for in-frame deletion of *mvfR*, Gm^R	This study
pEX18Gm-Δ*amb*	pEX18Gm with the fusion product of the *ambBCDE* flanking fragments for in-frame deletion of *amb* cluster, Gm^R	This study
pEX18Gm-Δ*pch*	pEX18Gm with the fusion product of the *pchABCDREFG* flanking fragments for in-frame deletion of *pch* cluster, Gm^R	40
mini-CTX-P*phz1*-lacZ	integration-proficient vector with the promoter region of *phzA1*, Tet^R	This study
mini-CTX-P*phz2*-lacZ	integration-proficient vector with the promoter region of *phzA2*, Tet^R	This study

Supplementary Table S2 Primers used in this study

Application	Oligos and sequence(5’to3’)
Deletion of *lasR*	*lasR*-F1: GGAATTCCAGCGTGGTGGTGTGCGAGATGTT
	lasR-R1: GTGCCCGACGACCCCTGAAGATGT
	lasR-F2: ggggtcgtcgggcacGCCTGGCGCGAGCATTACGAC
	lasR-R2: CGGGATCCCGCTACGCGGCGGGAGGTCAC
	lasR-con-F: TGGCCTTGGTTGACGGTTTTCTTG
Deletion of *rhlR*	*rhlR*-F1: GGAATTCCTGCCGCCGGTACACCCCAAGTTC
	rhlR-R1: CAACCCTATCTGTTATGCCAGCACCG
	rhlR-F2: taacagatagggttgTTCGACGCGCCGAACAAGACG
	rhlR-R2: CGGGATCCGTGCGCGAAACGGCTGACGACCT
	rhlR-con-F: GCTCGCGATTGGGGCCTCTGTGT
Deletion of *mvfR*	*mvfR*F1: GGAATTCCACGCGCCACCCAATAAAAGGAAT
	*mvfR*R1: CGGCGGGATGGCGGTGTC
	*mvfR*F2:accgccatcccgccgCGCGCCGCACCAGAGTAGAG
	*mvfR*R2: CCCAAGCTTGAGGCCGAGGAAACCCGCAAC
	mvfR-con-R AGCCGAGCACGCACTGGTTGAAGC
Deletion of *amb* cluster	*amb*F1: GGAATTCCGCGGCGTACTCCACCATCTGTTC
	*amb*R1: CGCCGCCAGTACCCCGCTCCATT
	*amb*F2: ggggtactggcggcgCCGGCGAACCTGGCAACCTGA
	*amb*R2: CCCAAGCTTGGCCGCCGCGAACGCTTAGAAAAA
	amb-con-F: CTTCCTCCGCCATGGGGTGTGCTA
	amb-con-R: CGGCCGTGGACGCTGATGAAGTT
Deletion of *pch* cluster	*pch*-F1:CGGAATTCGTTCCGAATCGCCTACCAGACCGC
	pch-R1:CAATGGGAAGCCAAGGTGAGCGAC
	pch-F2:cttggcttcccattgCCAGCTCACCCATCTCGACCAGGC
	pch-R2:ACGCGTCGACGAAACCGGCATACCAGTCGTCCCG
	pch-con-F:GCTGGCGCGGACGTTGTAGAGAT
	pch-con-R:TGCCGCCCGCCAATGATAATAAAT
Construction of *phz1* reporter strain	P*phz1*-For: GGAATTCCACATTTCCGTAACCCGAGAAGTACCC
	P*phz1*-Rev: CGGGATCCGGGTGTTTCCCTGTACCGCTGACC
Construction of *phz2* reporter strain	P*phz2*-For: GGAATTCCTCAACTCCAGCAACAAGGCGGAG
	P*phz2*-Rev: CGGGATCCCCCTTTCAACCGTTGGTACTCTCG

The sequences with underline are the introduced restriction sites.
